# Regadenoson as a pharmacologic stressor in cardiac magnetic resonance imaging in congenital and acquired pediatric heart disease: initial experience

**DOI:** 10.1186/1532-429X-17-S1-P211

**Published:** 2015-02-03

**Authors:** Cory V Noel, Ramkumar Krishnamurthy, Moffett Brady, Rajesh Krishnamurthy

**Affiliations:** Pediatric Cardiology, Baylor College of Medicine, Houston, TX USA; Radiology, Texas Children’s Hospital, Houston, TX USA; Pharmacology, Texas Children’s Hospital, Houston, TX USA

## Background

Adenosine has traditionally been utilized for coronary hyperemia during myocardial perfusion assessment in adults, however regadenoson has recently become more popular [1]. With improved survival of congenital heart disease (CHD), and increased diagnosis of acquired heart disease (AHD), there's an increasing need for assessment of myocardial perfusion in pediatrics [2]. Only dipyridamole and adenosine have reportedly been utilized as hyperemia stressors in CHD patients. As a selective A_2A_ agonist, regadenoson has a more favorable side-effect profile and less stimulation of receptors associated with bronchospasm, and decreased chronotropy [1]. It is administered as an intravenous (IV) bolus and thus only a single IV is required. Peak onset is at 60-90 seconds, with hyperemia lasting up to 6 minutes allowing additional wall motion assessment [3].

## Purpose

To observe the safety, feasibility and effectiveness of regadenoson as a pharmacologic stressor for perfusion CMR in a pilot cohort of CHD and pediatric AHD patients, as part of an ongoing.

## Methods

We reviewed our initial experience with regadenoson stress CMR in 9 subjects (aged 16.6 +/- 1.6 yrs) with CHD and pediatric AHD. All patients underwent stress CMR due to clinical symptoms and/or signs suggestive of ischemia. Cohort included 6 patients with D-TGA, 2 with coronary aneurysm, and 1 with coarctation.

### Acquisition protocol

See table for full details. Heart rate and blood pressure were monitored during CMR. Pharmacologic stress obtained by injecting 400 mcg of regadenoson 60 seconds prior to stress perfusion assessment. Rest and stress myocardial perfusion were assessed during the administration of 0.1 mmol/kg of gadolinium IV. Ventricular wall motion was assessed with cine SSFP imaging. Myocardial viability imaging was performed. Patients were monitored for 2 hours after the study.

### Data analysis

Myocardial perfusion and viability images were jointly assessed by a pediatric cardiologist and radiologist. MR images were assessed for WMA.

## Results

All stress CMR examinations were completed uneventfully. Peak stress was achieved in all studies. There were no serious adverse events. Resting heart rate was 63 +/- 12 beats per minute (bpm) and rose to a peak of 132 +/- 22 bpm.

**Figure 1:**
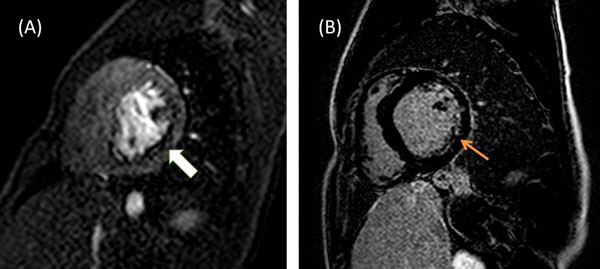
Basal short-axis slice demonstrating perfusion defect in the posterior and lateral myocardium consistent with circumflex artery occlusion(A). Corresponding myocardial viability. images demonstrated delayed-enhancement (B).

One patient with D-TGA had a fixed perfusion defect in the circumflex coronary artery distribution (fig 1), and one patient with D-TGA had reversible subendocardial perfusion defect in the left anterior descending distribution.

**Figure 2:**
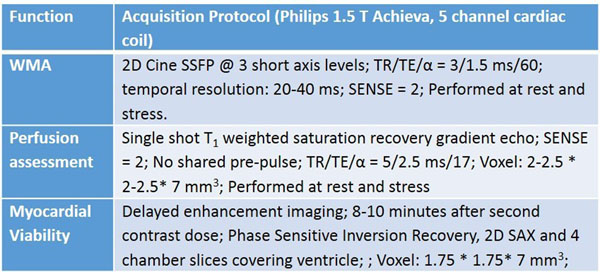
Acquisition Protocol used for wall motion analysis (WMA), perfusion assessment and myocardial viability is listed.

## Conclusions

Regadenoson may be a safe, feasible and effective pharmacologic stress agent for use in CMR of CHD and pediatric AHD. The use of a single IV bolus, and the advantage of a prolonged hyperemia makes it appealing in pediatrics.
